# Plant Diversity Impacts Decomposition and Herbivory via Changes in Aboveground Arthropods

**DOI:** 10.1371/journal.pone.0106529

**Published:** 2014-09-16

**Authors:** Anne Ebeling, Sebastian T. Meyer, Maike Abbas, Nico Eisenhauer, Helmut Hillebrand, Markus Lange, Christoph Scherber, Anja Vogel, Alexandra Weigelt, Wolfgang W. Weisser

**Affiliations:** 1 Institute of Ecology, University of Jena, Jena, Germany; 2 Department of Ecology and Ecosystem Management, Center for Food and Life Sciences Weihenstephan, Technische Universität München, Freising, Germany; 3 Institute for Chemistry and Biology of the Marine Environment, Carl-von-Ossietzky-University Oldenburg, Wilhelmshaven, Germany; 4 Max Planck Institute for Biogeochemistry, Jena, Germany; 5 DNPW, Agroecology, Georg-August University Göttingen, Göttingen, Germany; 6 Department for Systematic Botany and Functional Biodiversity, University of Leipzig, Leipzig, Germany; University of Tartu, Estonia

## Abstract

Loss of plant diversity influences essential ecosystem processes as aboveground productivity, and can have cascading effects on the arthropod communities in adjacent trophic levels. However, few studies have examined how those changes in arthropod communities can have additional impacts on ecosystem processes caused by them (e.g. pollination, bioturbation, predation, decomposition, herbivory). Therefore, including arthropod effects in predictions of the impact of plant diversity loss on such ecosystem processes is an important but little studied piece of information. In a grassland biodiversity experiment, we addressed this gap by assessing aboveground decomposer and herbivore communities and linking their abundance and diversity to rates of decomposition and herbivory. Path analyses showed that increasing plant diversity led to higher abundance and diversity of decomposing arthropods through higher plant biomass. Higher species richness of decomposers, in turn, enhanced decomposition. Similarly, species-rich plant communities hosted a higher abundance and diversity of herbivores through elevated plant biomass and C:N ratio, leading to higher herbivory rates. Integrating trophic interactions into the study of biodiversity effects is required to understand the multiple pathways by which biodiversity affects ecosystem functioning.

## Introduction

Extensive studies have identified biodiversity loss as one of the main drivers of ecosystem change [1], showing that the relation between biodiversity and ecosystem processes is mainly positive [2], [3]. For a long time biodiversity research has focused mainly on processes directly relating to the trophic level manipulated (e.g. aboveground productivity in plant diversity experiments [4], [5]; but see [6]). More recent studies have shown that plant diversity and associated changes in plant species composition also affect ecosystem processes governed by higher trophic levels, such as decomposition and herbivory [7]–[10].

Decomposition of organic matter is a key process in ecosystems, replenishing the pool of plant available soil nutrients and releasing photosynthetically fixed carbon back to the atmosphere by the activity of several groups of soil organisms. Invertebrate macrofauna, such as isopods, diplopods and earthworms, fragment dead plant material and thereby pave the way for further microbial decay and mineralization. Consequently, changes in their density and diversity should lead to altered decomposition rates [11], [12]. They may also affect nutrient cycling and plant productivity of grassland ecosystems. Herbivores can decrease plant productivity by decreasing plant performance, but contrary to that, they could also increase plant productivity by recycling nutrients and triggering compensatory growth [13], [14]. Arthropod density and diversity were discussed as drivers of plant diversity effects on decomposition and herbivory, but empirical evidence is scarce [8], [15]. Studies in experimental grasslands have generally reported higher diversity and abundances of herbivores, detritivores, pollinators, carnivores and parasitoid arthropods at higher plant diversity [16]–[23], although the strength of the effect differed between the groups.

**Figure 1:**
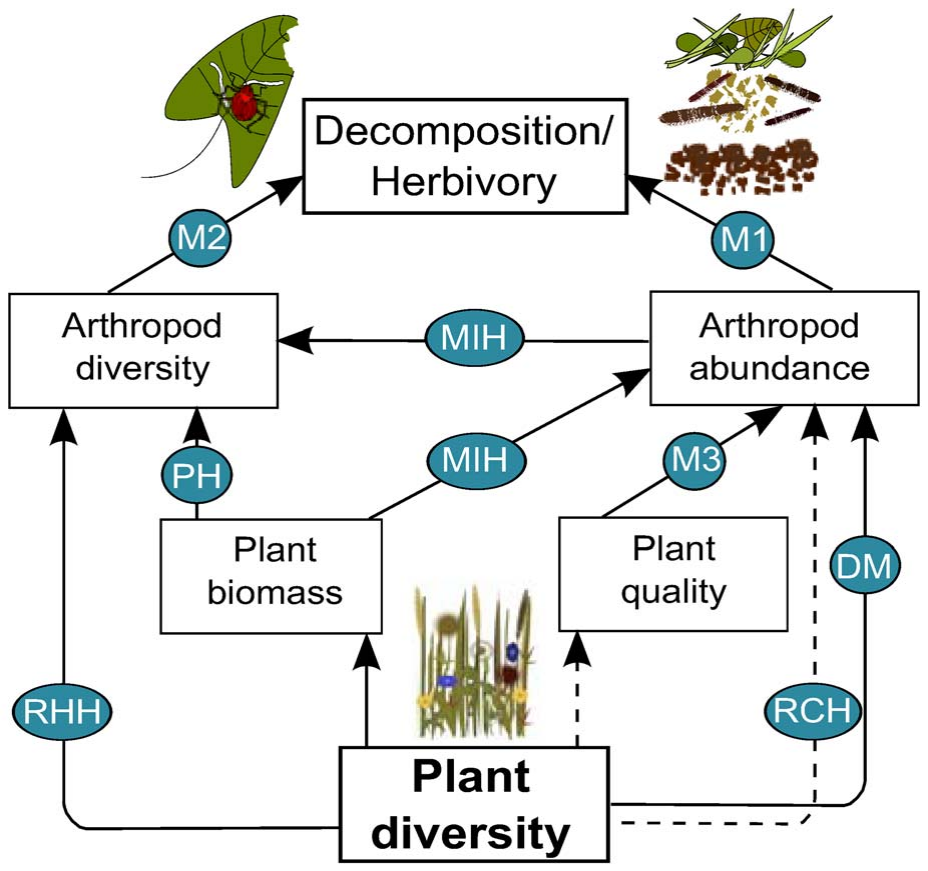
Possible mechanisms driving a positive plant diversity-ecosystem process relationship. The consumer-related ecosystem processes studied here are herbivory and decomposition. Based on existing literature we hypothesize different mechanisms driving the positive relationship between plant diversity and herbivory/decomposition, represented here by the different arrows. For details on the hypotheses see Introduction. Solid lines indicate expected positive correlations, dotted lines expected negative correlations. M1-3 = Mechanisms 1-3, DM = Dietary Mixing, MIH = More Individuals Hypothesis, PH = Productivity Hypothesis, RCH = Resource Concentration Hypothesis, RHH = Resource Heterogeneity Hypothesis. M1-3 are “unnamed” mechanisms, which are reported in the literature.

Plant-animal, plant-ecosystem process, and animal-ecosystem process relationships have often been studied separately, but have rarely been linked. To understand how plants, arthropods, and arthropod-related processes such as decomposition and herbivory are linked, there is a strong need to conduct studies considering multiple trophic levels and ecosystem processes simultaneously. There is a large number of competing hypotheses linking consumer-resource dynamics in diverse communities (summarized in Fig. 1), applicable to the relationship between arthropod and plant communities. Hypotheses emphasizing effects of resources on consumers have focused on biomass (More Individuals Hypothesis- MIH, Productivity Hypothesis- PH), and stoichiometry (C:N- Mechanism 3 in Fig. 1), both variables being strongly affected by changing plant species richness [24], [25] but also on species richness per se (Dietary Mixing- DM, Resource Concentration Hypothesis- RCH, Resource Heterogeneity Hypothesis- RHH).

MIH and PH hypothesize a positive relationship between plant biomass and consumer diversity, but they differ in the underlying mechanisms. The MIH [26] applies to communities, which are limited in productivity and predicts higher consumer abundances in more productive sites as well as consumer diversity as an increasing function of total abundance. Following the PH, a higher overall resource level in diverse plant communities directly attracts more generalist consumer species [27]. The stoichiometry of resources is also reported to be a strong predictor of consumer abundances as higher food quality (e.g. higher N content of plants) increases arthropod fitness (e.g. fecundity) and hence their abundance [28]. Following the literature, the way and direction how plant species richness affects consumer abundance strongly depends on their food specialization. According to the RCH [29] we would expect lower abundances of specialized herbivorous pests in more diverse habitats due to lower densities of the respective host plant. For generalist herbivores the opposite pattern may be true (DM): the ability of some generalist herbivores (e.g., grasshoppers) to mix their diet to an optimal combination of nutrients or to dilute toxins could increase their fitness and hence their abundance, indicated by a positive relation between plant diversity and arthropod abundance [30]. For consumer species richness the RHH [31] predicts that due to a higher number of different resources with increasing plant diversity, the number of specialist consumer species increases.

While the theoretical framework of how plant diversity affects consumer (arthropod) abundance and diversity is quite elaborated, the effects of arthropod communities on herbivory and decomposition are less clear. Consumer effects on related processes could be driven by arthropod abundance (Mechanism 1 in Fig. 1) or species richness (Mechanism 2 in Fig. 1). If specialist herbivores dominate, then increasing herbivore diversity is likely to increase community-wide herbivory rates, while a higher number of herbivores will only increase herbivory on those plants that are attacked. By contrast, if herbivory is mainly due to generalists, then increasing herbivore abundance rather than diversity will increase herbivory. For decomposition, functional complementarity among decomposers has been shown in a number of studies [11], yet decomposition may well be limited by decomposer abundance. Thus, various chains of effects are possible emphasizing the need for empirical approaches. All the hypotheses and mechanisms (Fig. 1) are not mutually exclusive, however, and many of the hypothesized pathways can have interactive and direct or indirect effects. The relative importance of these pathways may change when considering herbivores as opposed to decomposers because some mechanisms like compensatory plant growth would only result from herbivory, but not from decomposition.

Here, we integrate consumers into our models of plant diversity effects on ecosystem processes by focusing on three components of plant communities (food quantity: aboveground biomass; food quality: C:N ratio of plants, and species richness- hereafter: plant diversity), two components of consumer communities (arthropod abundance, arthropod species richness), and two processes (decomposition and herbivory). We test (1) if plant diversity effects on arthropods and decomposition/herbivory found in former studies can be confirmed, (2) if effects of plant diversity on decomposition and herbivory are mediated by altered arthropod community composition in terms of overall abundance, and (3) which mechanisms link plants to arthropods (plant diversity, quantity or quality), and arthropods to decomposition and herbivory (abundance or species richness).

## Material and Methods

### Ethic statement

Arthropod sampling was conducted with the permission of the city council of Jena, Germany.

### Experimental field site

The study was conducted in the framework of The Jena-Experiment (Thuringia, Germany, 50°55′ N, 11°35′ E; 130 m above sea level). This grassland biodiversity experiment uses sixty species native to regional floodplains. Plant communities were sown in 82 plots of 20×20 m with a gradient of species richness (1, 2, 4, 8, 16 and 60) and plant functional group richness (1–4 functional groups: grasses, small herbs, tall herbs, legumes) [32]. In 2009, the plot size was reduced to 100 m^2^, and two of the monocultures were abandoned due to poor cover of the target species. The particular species mixtures were randomly selected with some constraints [32]. A gradient in abiotic soil properties was taken into account by arranging plots in four blocks perpendicular to the river Saale [32]. All plots are mown twice a year, a management regime common for such meadows. Plots are weeded twice a year to maintain the experimental diversity gradient. As sown and realized number of target species are highly correlated (R^2^ = 0.97), we used the number of sown plant species for our analyses. The study site was established in May 2002 allowing for plenty of time for colonization by arthropods and making the established plant diversity gradient particularly well suited for examining consumer effects on ecosystem functioning [20].

### Plant community analyses

In our path analyses we used aboveground plant biomass as measure of resource availability for arthropods, because herbivores directly feed on plants and decomposers on plant litter material, which is reported to be highly correlated with plant biomass [19]. In 2010, aboveground plant community biomass was harvested during peak standing biomass in late May and August on all plots, in the frame of the long-term measurement of aboveground productivity. This was done by clipping the vegetation three cm above the soil surface in two rectangles of 0.2×0.5 m. The harvested biomass was separated into sown species, cleaned from weeds, dried at 70°C for 72 h and weighed. As a general measure of plant quality we used plant molar C:N ratio, because it is the most practicable measure of plant quality across 60 different plant species, applicable to many different herbivore and decomposer species. Community carbon (C) and nitrogen (N) concentrations of the pooled, dried and milled plant material were measured separately for May and August 2010 with an elemental analyzer (Flash EA 112 Thermo).

### Arthropod sampling

Arthropods were sampled in 2010 according to methods used in previous years with the permission of the city council of Jena. We sampled decomposers from May until September 2010, using two pitfall traps of 4.5 cm diameter per plot. The use of pit fall traps is quite common to sample litter- and surface-dwelling macro-arthropods like isopods and diplopods [33]. Traps were emptied every two weeks, but closed for two weeks during the mowing campaigns in June and September. In total, this resulted in 125 sampling days over the whole vegetation period. During the sampling periods, the field traps were filled with 3% formalin, and after emptying the traps, animals were stored in 70% ethanol. The resulting activity abundance data will be termed ‘abundance’ hereafter. Our data on decomposers comprise surface active decomposer macrofauna only. Less mobile decomposers, such as earthworms, may be important agents in surface litter decomposition [34], but are underrepresented in these samples.

Herbivorous arthropods were collected by suction sampling in June and July using a modified commercial vacuum cleaner (Kärcher A2500, Kärcher GmbH, Winnenden, Germany). Three subplots of 0.75 m×0.75 m were randomly chosen within each plot, covered quickly by a cage with gauze coat of the same size, and sampled until no arthropods were spotted anymore. The sampling was carried out between 9 a.m. and 4 p.m. within two 4-day sampling periods. Pit fall samples and suction samples were sorted to order level and most orders containing decomposers and herbivores were further identified to species level. For herbivores only Auchenorrhyncha, Coleoptera, and Heteroptera were identified to species level, and these orders covered 55% of the potential herbivores we sampled. Aphids and Diptera, which made together 30.2% of the sampled potential herbivores were not identified, because of their patchy distribution within a single plot (Aphids) and due to identification difficulties (Diptera).

Data of the different sampling campaigns were pooled.

### Litter decomposition

We measured litter decomposition using the standard litterbag technique [11]. We placed one litterbag in each of the 80 plots of The Jena-Experiment between 17^th^ June and 24^th^ August 2009, as successfully done in previous studies [9], [10], [35]. The fact that we have a very high number of replicates within each plant diversity level (across plots) compensates for the low replication within plots (one litterbag each) since we were more interested in the effects of plant diversity than of those of specific plant mixtures. To allow access for decomposer macrofauna (e.g. earthworms, isopods, diplopods) we constructed litterbags with 4 mm mesh and anchored them to the soil surface. We filled each litterbag with ∼3 g of wheat straw (N = 0.4%, C = 45.2%, C:N = 111.5) of ∼3 cm length. At the end of the experiment, the remaining litter was cleaned, dried (70°C, 48 h) and weighed to determine mass loss. The use of wheat straw acts as a standard method to measure potential decomposition rates caused by decomposers and affected by microclimatic conditions, without including effects of litter quality [9], [10], [36]. This method focusses on microenvironmental effects on decomposition caused by changes in plant communities instead of combined effects of microenvironment and litter quality, composition or structure.

### Herbivory

Arthropod herbivory was measured in the plant communities during peak biomass in August 2010. A maximum of 30 fully developed leaves were sampled randomly for each species from each plot where it occurred in the plant biomass samples. The minimum number of leaves for a species in a plot was one, and the average number was 21 per species and plot. On average 65% (SE±2.1%) of the sown species per plot occurred in the biomass sampled, therefore the measure can be regarded representative of the sown plant communities. The damaged surface area caused by herbivores of each leaf (in mm^2^) was estimated visually by comparing the damaged leaf area to circular and square templates ranging in size from 1 mm^2^ to 500 mm^2^. The leaf area was measured with a leaf-area meter (LI-3000C Area Meter, LI-COR Biosciences, Lincoln, USA), and the proportional herbivory damage (herbivory rates) was calculated as the damaged area divided by measured leaf area. The community herbivory rates for each plot were calculated by summing the species-specific herbivory rates weighted by species-specific leaf biomass in the plot. Mammals were excluded from the experimental field site by a fence, thus only invertebrates were responsible for the observed herbivore damage. Slugs contributed to the measured leaf damage, but were not included in herbivore analyses. For details in the methods see [8].

### Statistical analysis

We analyzed our data using (i) linear models and (ii) path analyses. Linear models served as affirmation of the results arising from the path analyses, as our sample size (N = 80) was somewhat lower than usually recommended for path analyses [37], [38].

Linear models were used to test for effects of plant community properties on arthropods and ecosystem processes. These models were fitted using the statistical software package R (R development Core Team, http://www.R-project.org. version 2.13.1). Arthropod abundance, arthropod species richness (decomposer and herbivores), decomposition and herbivory were used as response variables. For each of the response variables, we fitted a model including block (factor), plant diversity (numeric), and functional group richness (numeric; number of plant functional groups occurring in the respective plot- see experimental design section) as explanatory variables. We omitted plant functional group richness from our further models, as it had no consistent effect on any of the response variables (see Table S1).

Path analyses were calculated in Amos v.20.0 (IBM SPSS Amos, http://www.spss.com/amos/) and were used to infer the hypothesized causal links between plant diversity and ecosystem processes, as ecosystem process responses are likely indirectly mediated by physical, structural or chemical attributes of plants and/or consumer groups studied. We constructed two models (one for decomposition and one for herbivory), based on our hypotheses extracted from the published literature (see Introduction, and Fig. 1), and did not perform any model simplification. Models included arthropod abundance and species richness, aboveground plant biomass as a measure of food quantity, and plant community C:N ratio as measure of food quality as endogenous variables. Because of our low overall sample size (N = 80), we did not construct latent variables but assumed direct paths among each pair of endogenous variables. As herbivores were sampled after the first biomass harvest we used herbivory, biomass and C:N data of the data collection in August 2010. For decomposition we used mean values of the first and second data collection for aboveground biomass and C:N ratio of plants.

For all analyses (linear models and path analyses) measures of plant diversity, decomposer abundance, herbivore abundance and decomposer species richness were log transformed, aboveground plant biomass was square-root transformed, and herbivory was logit transformed [39] to account for non-normality of errors and heteroscedasticity, and to linearize the relationships among variables. All data used in this publication are deposited in the project database (www.the-jena-experiment.de).

## Results

### Effects of plant diversity on the decomposer community and decomposition

Overall, the final dataset contained 1,316 macrofauna decomposer individuals belonging to 12 species (four Isopoda and eight Diplopoda species). Isopoda dominated the macrofauna decomposer community, representing on average 92.8% of the total community.

Decomposer abundance and species richness increased significantly with increasing plant diversity, but remained unaffected by the number of plant functional groups (Table S1, Fig. S1). The number of individuals increased from 78.6 per plot (SE±19.2) in monocultures to 244 per plot (SE±30.5) in 60-plant-species-mixtures. Mean decomposer species richness increased from 3.6 (SE±0.5) per plot in plant monocultures to 6.3 (SE±0.4) in 60-plant-species-mixtures. Decomposition rate increased with increasing plant species richness from 3 mg mg^-1^ d^-1^ (SE±0.0002) in monocultures up to 5 mg mg^−1^ d^−1^ (SE±0.0004) in 60-plant-species-mixtures. The number of plant functional groups, available in the respective plant communities (Table S1), did not significantly affect decomposition.

### Mechanisms driving altered decomposition rates

**Figure 2:**
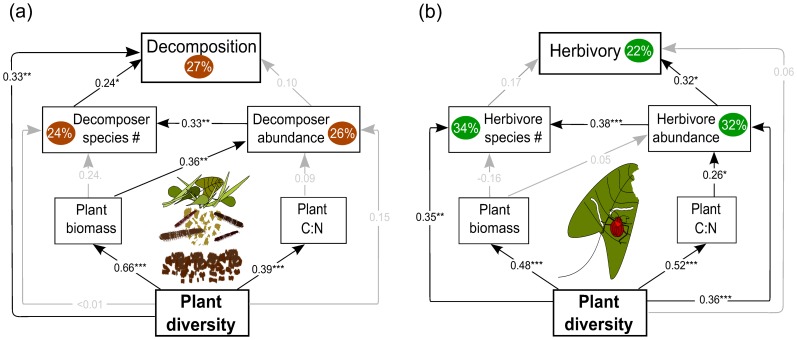
Path diagram explaining plant community effects on decomposition and herbivory. Models relate plant community variables (diversity, quantity and quality), species richness and abundance of (a) decomposer arthropods to decomposition, and (b) herbivorous insects to herbivory. Standardised path coefficients are given on top of the path arrows with significances indicated by *, *P*<0.05; **, *P*<0.01; ***, *P*<0.001. Non-significant paths are given in grey.

**Figure 3:**
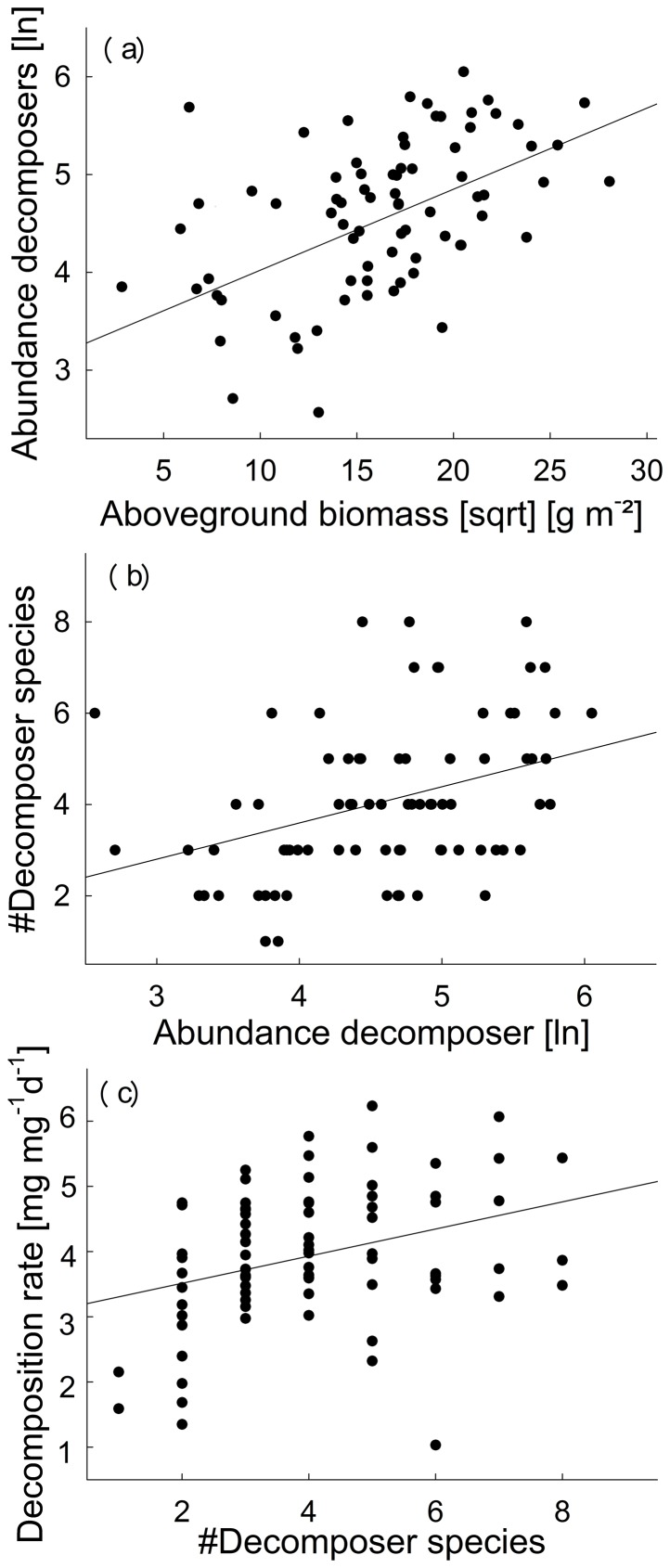
Pairwise correlations visualizing the significant links detected in the path analysis relating plants, decomposers and decomposition. We show the relationships between aboveground plant biomass and decomposer abundances (a), between decomposer abundances and their species richness (b) and decomposer species richness and decomposition (c). For statistics, see Table S2.

A chi-squared test indicated that our path analysis model cannot be rejected as a potential explanation of the observed covariance matrix (*χ^2^_4_* = 4.49, *P* = 0.344). The model explained 27% of the variance in decomposition. The positive effect of plant diversity on decomposition (Table S2 and S3, Fig. 2a) was mediated by aboveground plant biomass, which increased with increasing plant diversity and which positively affected decomposer abundance (Fig. 3a) and species richness (marginally). Although decomposer abundances and species richness were strongly correlated (Fig. 3b; Table S1), only species richness increased litter decomposition (Fig. 3c; Table S1). While increasing plant diversity increased plant C:N ratio, there was no significant effect of plant C:N ratio on decomposers or decomposition. A chi-squared test for the same path analysis excluding species richness and abundance of decomposers indicated that it need to be rejected as a potential explanation of the observed covariance matrix (*χ^2^_6_* = 15.60, *P* = 0.016).

### Effects of plant diversity on the herbivore community and herbivory rate

Overall, the final dataset contained 12,829 herbivores, belonging to 127 species (38 Auchenorrhyncha, 62 Coleoptera, 27 Heteroptera). On average the herbivore community was dominated by Coleoptera (48.6%), followed by Auchenorrhyncha (43.6%), and Heteroptera (7.8%). Plant diversity, but not plant functional group richness, strongly affected the abundance and species richness of herbivorous insects and herbivory rate (Table S1, Fig. S2). Herbivore abundance increased from 65.2 (SE±13.7) individuals per plot in monocultures to 251 (SE±36.7) individuals per plot in 60-plant-species-mixtures. Herbivore communities in monocultures contained on average 15.6 (SE±1.0) species per plot, whereas in 60-plant-species-mixtures 23.5 (SE±0.8) species per plot occurred. Plant diversity also led to a higher herbivory rate, nearly doubling from 2% (SE±0.4%) in monocultures to 3.5% (SE±0.9%) in 60-plant-species-mixtures.

### Mechanisms driving altered herbivory

**Figure 4:**
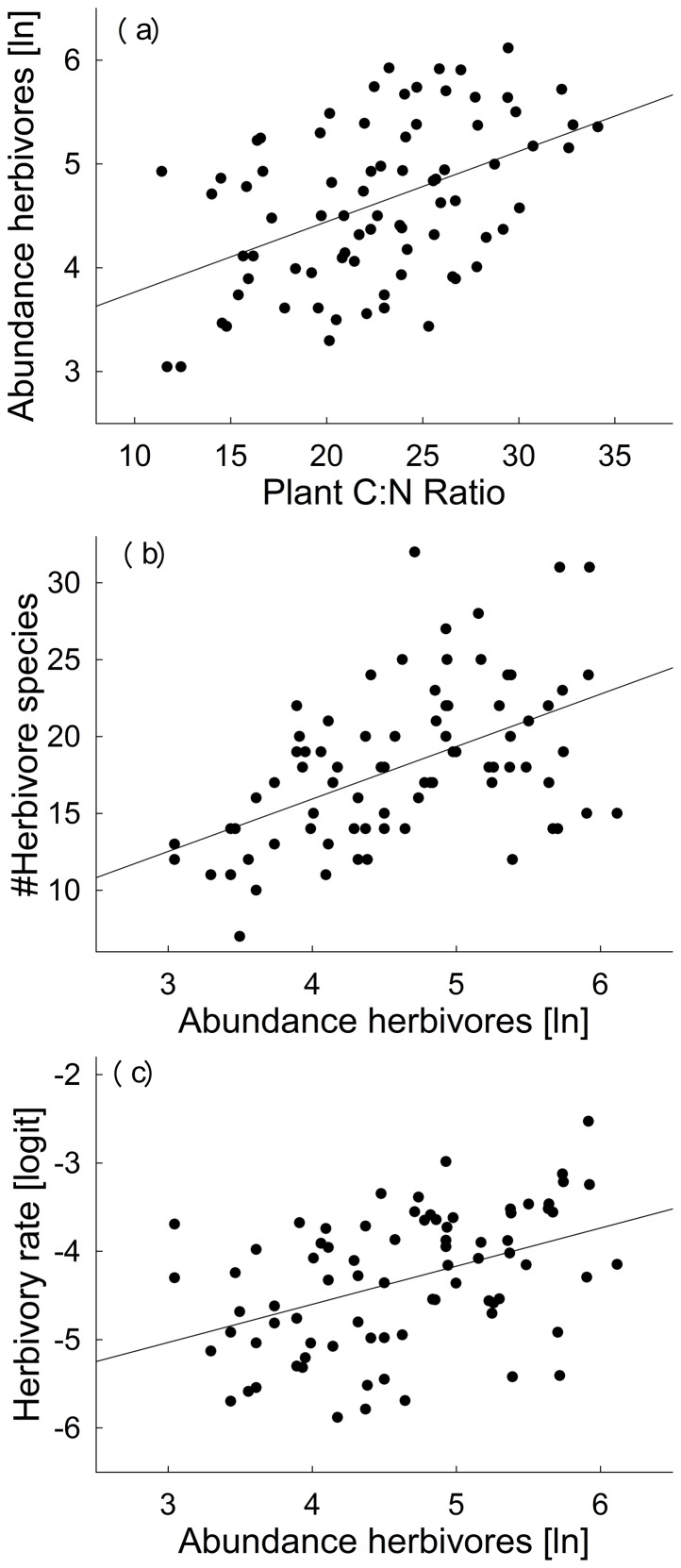
Pairwise correlations visualizing the significant links detected in the path analysis relating plants, herbivores and herbivory. We show the relationships between plant C:N ratio and herbivore abundance (a), herbivore species richness and their abundance (b), and between herbivore abundance and herbivory rate (c). For statistics, see Table S4.

A chi-squared test indicated that the path analysis explained 22% of the variance in herbivory, and cannot be rejected as a potential explanation of the observed covariance matrix (*χ^2^_4_* = 7.50, *P* = 0.112). The positive relationship between plant diversity and herbivory rate (Table S4 and S5, Fig. 2b), was not driven by aboveground plant biomass, as there was no significant correlation between aboveground biomass and herbivore abundance and species richness. Herbivore abundance and species richness increased significantly with increasing plant diversity (Fig. S2). Increasing plant diversity increased plant C:N ratio, which, in turn, was positively correlated with herbivore abundance (Fig. 4a). Herbivore abundances increased herbivore species richness (Fig. 4b), but only higher abundances of herbivorous arthropods increased herbivory rate (Fig. 4c). A chi-squared test for the same path analysis excluding species richness and abundance of herbivores indicated that it need to be rejected as a potential explanation of the observed covariance matrix (*χ^2^_6_* = 24.70, *P* = 0.001).

## Discussion

Plant diversity influenced the abundance and diversity of decomposers and herbivores, and in turn, these effects propagated to decomposition and herbivory rates. Effects of plant diversity on process rates could be better explained when including information on the consumer community into the explanatory model for both, decomposition and herbivory. Abundance of decomposers was positively associated with increased resource (plant) biomass, whereas herbivore abundance increased with higher C:N ratio of the plant material and plant species richness. However, herbivore richness increased directly with higher plant diversity, whereas decomposer richness increased indirectly (via plant biomass and decomposer abundance). Accordingly, we provide support for the More Individuals Hypothesis (MIH) to drive altered decomposer communities, whereas we explain changes in herbivore communities by Dietary Mixing (DM) and the Resource Heterogeneity Hypothesis (RHH). Decomposer species richness enhanced decomposition of litter material (Mechanism 2 in Fig. 1), whereas higher herbivory was mainly driven by the higher abundance of herbivores (Mechanism 1 in Fig. 1).

### Decomposers and decomposition

Notably, the discussion of plant community effects (biomass and C:N ratio) on the decomposer community is based on to the use of standard litter material in our study instead of plot-specific litter material. Consequently, we here discuss indirect effects between plant variables and decomposer communities (plants affect the environment for decomposers, which in turn leads to changes in the decomposer community) [9], [10].

Decomposition was positively correlated with plant diversity as has been shown in some studies using standard litter [9], [40]–[42] but not all [10], [34]. Reich and colleagues [3] recently showed that plant diversity effects on plant biomass production increased over time, and it was suggested that this increase in plant diversity effects may be linked to delayed soil feedback effects of slowly assembling decomposer communities [43]. Indeed, the density and diversity of macro-decomposers and the density of meso-decomposers increased significantly with increasing plant diversity only four (aboveground) and six years (belowground) after establishment of The Jena-Experiment [20], [23]. Consequently, the lack of plant diversity effects in other studies may be due to the short-term character of most previous experiments. Positive effects of plant diversity on decomposition processes can possibly be attributed to higher productivity of more diverse plant communities and thus elevated food availability for decomposers (see MIH in Fig. 1), as the amount of available litter material is positively correlated to aboveground biomass in grasslands [44].

Our path analysis identified aboveground biomass as an important driver of changes in the decomposer abundance, which supports the MIH. Plant biomass production increased with increasing plant diversity and this, in turn, increased decomposer species richness via abundance. Resulting increases in decomposition rates were mediated by increased decomposer species richness but not abundance, indicating a more diverse decomposer community to be functionally complementary in fragmenting litter material (according to M2 in Fig. 1).

Our results on decomposition are in accordance with a study of Heemsbergen et al. [36], who found functional dissimilarity among decomposer species being the best predictor for changes in leaf litter mass loss and soil respiration. Similar results are reported for benthic detritivores in streams, where decomposition rates also increase with detritivore richness [45]. In these systems, potential mechanisms involve increasing faciliation of resource use among species and reduction of inter-specific competiton [46]. Such facilitation can arise if the processing of dead organic matter by one species enhances the suitability of this resource for another species. The positive correlation between decomposer species richness and decomposition could also occur due to a sampling effect (higher chance of having a functionally dominant decomposer species in a species-rich community than in a species-poor one); however, we are not able to disentangle both possible effects here.

Even if we used wheat straw as litter material in our study we expected a negative effect of plant C:N ratio on the environment for decomposers (lower nutrient availability in litter and soil), thereby indirectly affecting their fitness (in our case: abundance). In contrast to our hypothesis we detected no significant effect of aboveground plant quality (C:N ratio) on decomposer abundance and species richness, maybe indicating that the C:N ratio of the fresh plant material is a poor predictor of the C:N ratio of the litter material.

In our path analysis a significant direct link between plant diversity and decomposition remained, indicating that part of the plant diversity effect on decomposition was independent of consumer-mediated effects. Potential environmental variables that could contribute to this link might include soil moisture of the top soil layer, vegetation density, and shade which are important predictors of soil dwelling decomposer community composition. All three variables are documented to increase with increasing plant diversity [10], [47]. Further, our analysis was restricted to aboveground living decomposers, but we know that belowground decomposer groups are strongly affected by plant diversity [19], [48]. Therefore, we suggest that also belowground decomposition might show an imprint of plant species richness via decomposer abundance or diversity. As data on soil dwelling decomposer species were not available from the the study year and could therefore not be included in the analyses this is a natural extension for future studies.

### Herbivores and herbivory

Plant diversity increased both herbivore abundance and richness. Plant diversity effects on herbivore richness could not be explained by variations in plant biomass or plant C:N, which indicates that it may be a direct link between resource diversity and consumer diversity. Such relationships are expressly predicted for specialist consumers, as more diverse resource assemblages enable the presence of a higher number of specialist consumer species (e.g. [16], [17], [21], [31]; see RHH in Fig. 1). However, generalist consumers can also benefit from more diverse food plants, if these provide a nutritionally more balanced or temporally less variable food [17], [30]. In contrast to findings of McNaughton et al. [49] and Borer et al. [22], who found higher aboveground plant biomass at higher plant species richness providing the food for a larger abundance of herbivores (see MIH in Fig. 1), increases in herbivore abundance in this study were not mediated by plant biomass.

Food plant quality (here C:N ratio) affected the herbivore abundance and thereby indirectly herbivory. As previously shown [25], [50], plant C:N ratios increased with plant species richness. A higher food C:N ratio should lead to lower fitness and abundance of herbivores [28], [51], [52] if the plant C:N is above the consumers' threshold elemental ratios [53]. Consequently, community-wide herbivory was shown to decrease with poorer autotroph quality although individuals compensated for poor food quality by ingesting more [54]. If compensatory feeding in a low nutrition environment is effective, a neutral relationship between food quality and community herbivory might occur [51], [55]. Yet, it is hard to imagine how compensatory feeding could lead to increased herbivore abundance at higher plant C:N as observed in our analysis, unless C:N ratio is correlated with other non-nutritional aspects of these trophic interactions. Increased investment in vertical growth and thus C-rich stem-material in the more species-rich plots is a promising candidate for such a relationship, which would increase the habitable volume for arthropods and thereby increase abundance. This interpretation is supported by the fact that the average height in summer of all plants occuring in a communitiy was a significant predictor of herbivory levels when modelling community herbivory based on plant functional traits [56].

Overall, plant diversity increased both herbivore abundance and richness, but only herbivore abundance then increased herbivory rates (confirming M1 in Fig. 1). The obvious interpretation of this relationship is that at higher plant diversity a larger abundance of herbivores consumes more plant biomass. Consequently, an increase of herbivory at higher plant diversity [8], [57], can potentially be explained by an increase in herbivore abundance with increasing plant diversity, as documented in this study. Integrating data on feeding type or food specialization could help for a detailed understanding of the mechanisms responsible for the observed positive relation between herbivore abundance and herbivory rate. Herbivore species richness was indirectly linked to herbivory rates via its positive correlation to herbivore abundance.

### Conclusion

Our results demonstrate the importance of interactions across trophic levels for ecosystem process rates of green and brown food webs. Decomposition was driven by arthropod species richness via arthropod abundance, and herbivory depended on the number of herbivores. Excluding data on consumer levels from our analysis (thus measuring only the directly plant related links from plant diversity and composition to rates of decomposition or herbivory) resulted in no explained variance (model rejection, see result section) for decomposition and herbivory. In particular, our results strongly support the conclusion that integrating trophic interactions into the study of biodiversity effects is required to understand the multiple pathways by which biodiversity affects ecosystem functioning [58]. In addition, we could show that the functional role of an important part of overall biodiversity – in our case the arthropod community – cannot always be deduced *a priori*, but must be unraveled using a mechanistic approach. While functional biodiversity research has shown the general importance of biodiversity for many ecosystem processes, understanding the functional role of biodiversity requires analyzing the mechanisms underlying BEF relationships. Given that decomposers and herbivores interact with communities of natural enemies exerting a top-down control in addition to the bottom-up control by the plant community [59] a consequent step forward would be to include higher trophic levels (predators, parasites, parasitoids) in the analysis of plant diversity effects on arthropod-mediated ecosystem processes.

## Supporting Information

Figure S1 **Effects of plant diversity on the abundance and species richness of decomposer arthropods, and decomposition.**
(DOCX)

Figure S2 **Effects of plant diversity on the abundance and species richness of herbivorous arthropods, and herbivory rate.**
(DOCX)

Table S1 **Results of linear models testing plant diversity effects on decomposing and herbivorous arthropods, and decomposition (mg mg^−1^ d^−1^) and herbivory rate (%, logit transformed).**
(DOCX)

Table S2 **Results of the path analysis linking plants, decomposers and decomposition.**
(DOCX)

Table S3 **Standardized total effects of the structural equation model analysing plant diversity effects on decomposition.**
(DOCX)

Table S4 **Results of the path analysis linking plants, herbivores and herbivory rate.**
(DOCX)

Table S5 **Standardized total effects of the structural equation model analysing plant diversity effects on herbivory rate.**
(DOCX)

## References

[1] Hooper DU, Adair EC, Cardinale BJ, Byrnes JEK, Hungate BA, et al (2012) A global synthesis reveals biodiversity loss as a major driver of ecosystem change. Nature 486: 105–108. 22678289 10.1038/nature11118

[2] Cardinale BJ, Duffy JE, Gonzalez A, Hooper DU, Perrings C, et al (2012) Biodiversity loss and its impact on humanity. Nature 486: 59–67. 22678280 10.1038/nature11148

[3] Reich PB, Tilman D, Isbell F, Mueller K, Hobbie SE, et al (2012) Impacts of biodiversity loss escalate through time as redundancy fades. Science (80-) 336: 589–592. 10.1126/science.1217909 22556253

[4] Cardinale BJ, Srivastava DS, Duffy JE, Wright JP, Downing AL, et al (2006) Effects of biodiversity on the functioning of trophic groups and ecosystems. Nature 443: 989–992. 17066035 10.1038/nature05202

[5] Marquard E, Weigelt A, Temperton VM, Roscher C, Schumacher J, et al (2009) Plant species richness and functional composition drive overyielding in a six-year grassland experiment. Ecology 90: 3290–3302. 20120799 10.1890/09-0069.1

[6] Naeem S, Thompson LJ, Lawler SP, Lawton JH, Woodfin RM (1994) Declining biodiversity can alter the performance of ecosystems. Nature 368: 734–737.

[7] Scherber C, Milcu A, Partsch S, Scheu S, Weisser WW (2006) The effects of plant diversity and insect herbivory on performance of individual plant species in experimental grassland. J Ecol 94: 922–931.

[8] Loranger H, Weisser WW, Ebeling A, Eggers T, Luca E De, et al.. (2013) Invertebrate herbivory increases along an experimental gradient of grassland plant diversity. Oecologia:10.1007/s00442-013-2741-5 23907703

[9] Hector A, Beale AJ, Minns A, Otway SJ, Lawton JH (2000) Consequences of the reduction of plant diversity for litter decomposition: effects through litter quality and microenvironment. Oikos 90: 357–371.

[10] Scherer-Lorenzen M (2008) Functional diversity affects decomposition processes in experimental grasslands. Funct Ecol 22: 547–555.

[11] Hattenschwiler S, Gasser P (2005) Soil animals alter plant litter diversity effects on decomposition. Proc Natl Acad Sci U S A 102: 1519–1524. 15671172 10.1073/pnas.0404977102 PMC547817

[12] Eisenhauer N (2012) Aboveground-belowground interactions as a source of complementarity effects in biodiversity experiments. Plant Soil 351: 1–22.

[13] Gruner DS, Smith JE, Seabloom EW, Sandin SA, Ngai JT, et al (2008) A cross-system synthesis of consumer and nutrient resource control on producer biomass. Ecol Lett 11: 740–755. 18445030 10.1111/j.1461-0248.2008.01192.x

[14] Allan E, Crawley MJ (2011) Contrasting effects of insect and molluscan herbivores on plant diversity in a long-term field experiment. Ecol Lett 14: 1246–1253. 22017580 10.1111/j.1461-0248.2011.01694.x

[15] Mulder CPH, Koricheva J, Huss-Danell K, Hogberg P, Joshi J (1999) Insects affect relationships between plant species richness and ecosystem processes. Ecol Lett 2: 237–246.

[16] Koricheva J, Mulder CPH, Schmid B, Joshi J, Huss-Danell K (2000) Numerical responses of different trophic groups of invertebrates to manipulations of plant diversity in grasslands. Oecologia 125: 271–282. 24595838 10.1007/s004420000450

[17] Ebeling A, Klein AM, Schumacher J, Weisser WW, Tscharntke T (2008) How does plant richness affect pollinator richness and temporal stability of flower visits? Oikos 117: 1808–1815.

[18] Haddad NM, Crutsinger GM, Gross K, Haarstad J, Knops JMH, et al (2009) Plant species loss decreases arthropod diversity and shifts trophic structure. Ecol Lett 12: 1029–1039. 19702636 10.1111/j.1461-0248.2009.01356.x

[19] Scherber C, Eisenhauer N, Weisser WW, Schmid B, Voigt W, et al (2010) Bottom-up effects of plant diversity on multitrophic interactions in a biodiversity experiment. Nature 468: 553–556. 20981010 10.1038/nature09492

[20] Eisenhauer N, Milcu A, Sabais A, Bessler H, Brenner J, et al (2011) Plant diversity surpasses plant functional groups and plant productivity as driver of soil biota in the long term. PLoS One 6: e16055. 21249208 10.1371/journal.pone.0016055 PMC3017561

[21] Sabais ACW, Scheu S, Eisenhauer N (2011) Plant species richness drives the density and diversity of Collembola in temperate grassland. Acta Oecologica-International J Ecol 37: 195–202.

[22] Borer ET, Seabloom EW, Tilman D, Novotny V (2012) Plant diversity controls arthropod biomass and temporal stability. Ecol Lett 15: 1457–1464. 23020194 10.1111/ele.12006

[23] Rzanny M, Voigt W (2012) Complexity of multitrophic interactions in a grassland ecosystem depends on plant species diversity. J Anim Ecol 81: 614–627. 22292705 10.1111/j.1365-2656.2012.01951.x

[24] Cardinale BJ, Wright JP, Cadotte MW, Carroll IT, Hector A, et al (2007) Impacts of plant diversity on biomass production increase through time because of species complementarity. Proc Natl Acad Sci U S A 104: 18123–18128. 17991772 10.1073/pnas.0709069104 PMC2084307

[25] Abbas M, Ebeling A, Oelmann Y, Ptacnik R, Roscher C, et al (2013) Biodiversity effects on plant stoichiometry. PLoS One 8: e58179. 23483990 10.1371/journal.pone.0058179 PMC3587429

[26] Srivastava DS, Lawton JH (1998) Why more productive sites have more species: An experimental test of theory using tree-hole communities. Am Nat 152: 510–529. 18811361 10.1086/286187

[27] Abrams PA (1995) Monotonic or unimodal diversity productivity gradients - what does competition theory predict. Ecology 76: 2019–2027.

[28] Awmack CS, Leather SR (2002) Host plant quality and fecundity in herbivorous insects. Annu Rev Entomol 47: 817–844. 11729092 10.1146/annurev.ento.47.091201.145300

[29] Root RB (1973) Organization of a plant-arthropod association in simple and diverse habitats - fauna of collards (Brassica-Oleracea). Ecol Monogr 43: 95–120.

[30] Bernays EA, Bright KL (1993) Mechanisms of dietary mixing in grasshoppers - a review. Comp Biochem Physiol a-Physiology 104: 125–131.

[31] Hutchinson GE (1959) Homage to Santa-Rosalia or why are there so many kinds of animals. Am Nat 93: 145–159.

[32] Roscher C, Schumacher J, Baade J, Wilcke W, Gleixner G, et al (2004) The role of biodiversity for element cycling and trophic interactions: an experimental approach in a grassland community. Basic Appl Ecol 5: 107–121.

[33] Coleman DC, Crossley DA, Hendrix PF (2004) Fundamentals in soil ecology. Elsevier Academic Press.

[34] Milcu A, Partsch S, Scherber C, Weisser WW, Scheu S (2008) Earthworms and legumes control litter decomposition in a plant diversity gradient. Ecology 89: 1872–1882. 18705374 10.1890/07-1377.1

[35] Vogel A, Eisenhauer N, Weigelt A, Scherer-Lorenzen M (2013) Plant diversity does not buffer drought effects on early-stage litter mass loss rates and microbial properties. Glob Chang Biol 19: 2795–2803. 23606531 10.1111/gcb.12225

[36] Heemsbergen DA, Berg MP, Loreau M, van HajJR, Faber JH, et al (2004) Biodiversity effects on soil processes explained by interspecific functional dissimilarity. Science (80-) 306: 1019–1020. 10.1126/science.1101865 15528441

[37] Arbuckle JL, Wothke W (1995) Amos 4.0 User's guide. Chicago: Small waters.

[38] Grace J (2006) Structural equation modeling and natural systems. Cambridge: Cambridge University Press.

[39] Warton DI, Hui FKC (2011) The arcsine is asinine: the analysis of proportions in ecology. Ecology 92: 3–10. 21560670 10.1890/10-0340.1

[40] Bardgett RD, Shine A (1999) Linkages between plant litter diversity, soil microbial biomass and ecosystem function in emperate grasslands. Soil Biol Biochem 31: 317–321.

[41] Spehn EM, Hector A, Joshi J, Scherer-Lorenzen M, Schmid B, et al (2005) Ecosystem effects of biodiversity manipulations in european grasslands. Ecol Monogr 75: 37–63.

[42] Cardinale BJ, Matulich KL, Hooper DU, Byrnes JE, Duffy E, et al (2011) The functional role of producer diversity in ecosystems. Am J Bot 98: 572–592. 21613148 10.3732/ajb.1000364

[43] Eisenhauer N, Reich PB, Scheu S (2012) Increasing plant diversity effects on productivity with time due to delayed soil biota effects on plants. Basic Appl Ecol 13: 571–578.

[44] Long ZT, Mohler CL, Carson WP (2003) Extending the resource concentration hypothesis to plant communities: Effects of litter and herbivores. Ecology 84: 652–665.

[45] Jonsson M, Malmqvist B (2000) Ecosystem process rate increases with animal species richness: evidence from leaf-eating, aquatic insects. Oikos 89: 519–523.

[46] Jonsson M, Malmqvist B (2003) Mechanisms behind positive diversity effects on ecosystem functioning: testing the facilitation and interference hypotheses. Oecologia 134: 554–559. 12647128 10.1007/s00442-002-1148-5

[47] Rosenkranz S, Wilcke W, Eisenhauer N, Oelmann Y (2012) Net ammonification as influenced by plant diversity in experimental grasslands. Soil Biol Biochem 48: 78–87.

[48] Hooper DU, Bignell DE, Brown VK, Brussaard L, Dangerfield JM, et al (2000) Interactions between aboveground and belowground biodiversity in terrestrial ecosystems: Patterns, mechanisms, and feedbacks. Bioscience 50: 1049–1061.

[49] McNaughton SJ, Oesterheld M, Frank DA, Williams KJ (1989) Ecosystem-level patterns of primary productivity and herbivory in terrestrial habitats. Nature 341: 142–144. 2779651 10.1038/341142a0

[50] Van Ruijven J, Berendse F (2005) Diversity-productivity relationships: initial effects, long-term patterns, and underlying mechanisms. Proc Natl Acad Sci U S A 102: 695–700. 15640357 10.1073/pnas.0407524102 PMC545547

[51] Berner D, Blanckenhorn WU, Korner C (2005) Grasshoppers cope with low host plant quality by compensatory feeding and food selection: N limitation challenged. Oikos 111: 525–533.

[52] Ebeling A, Allan E, Heimann J, Köhler G, Scherer-Lorenzen M, et al (2013) The impact of plant diversity and fertilization on fitness of a generalist. Basic Appl Ecol 14: 246–254.

[53] Frost PC, Benstead JP, Cross WF, Hillebrand H, Larson JH, et al (2006) Threshold elemental ratios of carbon and phosphorus in aquatic consumers. Ecol Lett 9: 774–779. 16796566 10.1111/j.1461-0248.2006.00919.x

[54] Hillebrand H, Borer ET, Bracken MES, Cardinale BJ, Cebrian J, et al (2009) Herbivore metabolism and stoichiometry each constrain herbivory at different organizational scales across ecosystems. Ecol Lett 12: 516–527. 19392711 10.1111/j.1461-0248.2009.01304.x

[55] Schuldt A, Bruelheide H, Durka W, Eichenberg D, Fischer M, et al (2012) Plant traits affecting herbivory on tree recruits in highly diverse subtropical forests. Ecol Lett 15: 732–739. 22548792 10.1111/j.1461-0248.2012.01792.x

[56] Loranger J, Meyer ST, Shipley B, Kattge J, Loranger H, et al (2013) Predicting invertebrate herbivory from plant traits: polycultures show strong nonadditive effects. Ecology 94: 1499–1509. 23951710 10.1890/12-2063.1

[57] Schuldt A, Baruffol M, Böhnke M, Bruelheide H, Härdtle W, et al (2010) Tree diversity promotes insect herbivory in subtropical forests of south-east China. J Ecol 98: 917–926. 20852667 10.1111/j.1365-2745.2010.01659.x PMC2936109

[58] Duffy JE, Cardinale BJ, France KE, McIntyre PB, Thebault E, et al (2007) The functional role of biodiversity in ecosystems: incorporating trophic complexity. Ecol Lett 10: 522–538. 17498151 10.1111/j.1461-0248.2007.01037.x

[59] Rzanny M, Kuu A, Voigt W (2013) Bottom-up and top-down forces structuring consumer communities in an experimental grassland. Oikos 122: 967–976.

