# Supplementary material for: Plant Diversity Impacts Decomposition and Herbivory via Changes in Aboveground Arthropods
Source: PLoS One. 2014 Sep 16;9(9):e106529. doi: 10.1371/journal.pone.0106529 (PMC4165753; doi:10.1371/journal.pone.0106529)
Supplement: Figure S1 — Effects of plant diversity on the abundance and species richness of decomposer arthropods, and decomposition. (DOCX) [file pone.0106529.s001.docx]

**Figure S1:** Effects of plant diversity on the abundance and species richness of decomposer arthropods, and decomposition. We show the relationships between plant species richness and (a) decomposer abundance, (b) decomposer species richness, and (c) decomposition. For statistics, see Table S1. Dots give mean values per plot.

**
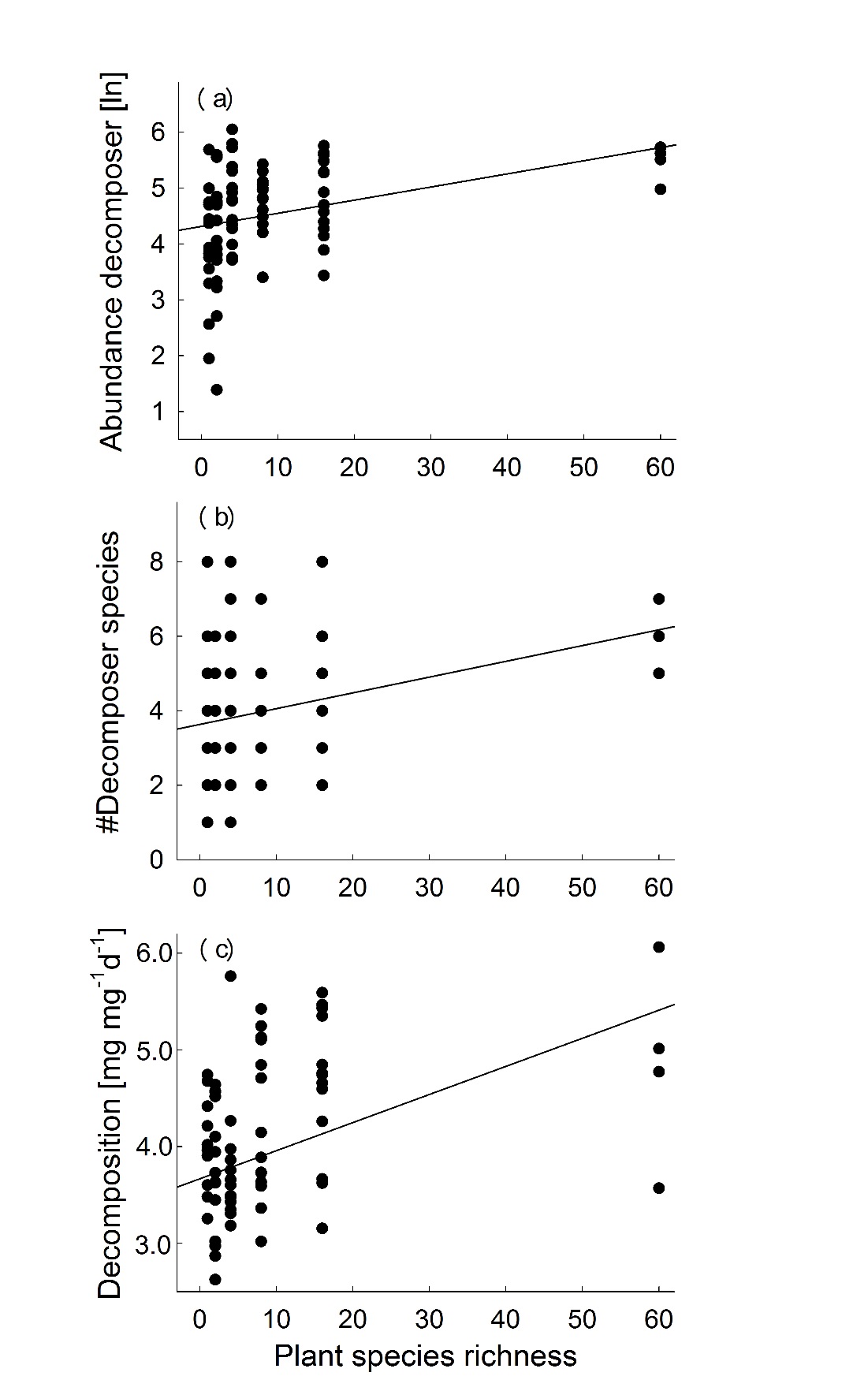
**
