# Supplementary material for: Plant Diversity Impacts Decomposition and Herbivory via Changes in Aboveground Arthropods
Source: PLoS One. 2014 Sep 16;9(9):e106529. doi: 10.1371/journal.pone.0106529 (PMC4165753; doi:10.1371/journal.pone.0106529)

**Figure S2:** Effects of plant diversity on the abundance and species richness of herbivorous arthropods, and herbivory rate. We show the relationships between plant species richness and (a) herbivore abundance, (b) herbivore species richness, and (c) herbivory rate. For statistics, see Table S1. Dots give mean values per plot.


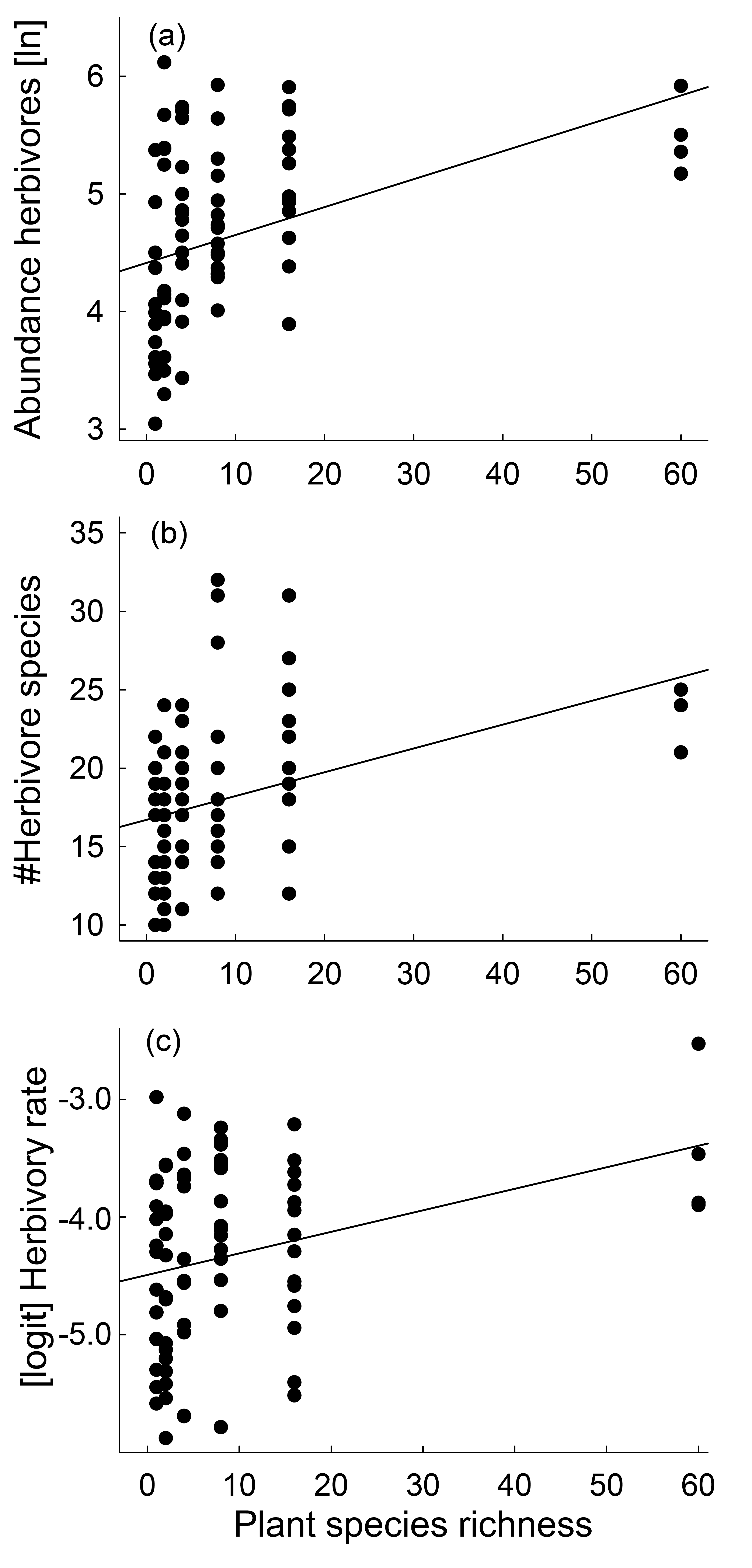

Supplement: Figure S2 — Effects of plant diversity on the abundance and species richness of herbivorous arthropods, and herbivory rate. (DOCX) [file pone.0106529.s002.docx]
