# Supplementary material for: Plant Diversity Impacts Decomposition and Herbivory via Changes in Aboveground Arthropods
Source: PLoS One. 2014 Sep 16;9(9):e106529. doi: 10.1371/journal.pone.0106529 (PMC4165753; doi:10.1371/journal.pone.0106529)
Supplement: Table S1 — Results of linear models testing plant diversity effects on decomposing and herbivorous arthropods, and decomposition (mg mg−1 d−1) and herbivory rate (%, logit transformed). (DOCX) [file pone.0106529.s003.docx]

**Table S1:** Results of linear models with the response variables block, plant diversity (log transformed), and plant functional group richness in relation to decomposition (mg mg^-1^ d^-1^) and herbivory rate (%, logit transformed) and abundance and richness of decomposing and herbivorous arthropods (log transformed). Statistics for all response variables are presented, and significant variables are given in bold.

| **Response variable** | **Block** | **Plant diversity** | **Functional group richness** |
| --- | --- | --- | --- |
| Decomposer abundance | **F_3,74_= 5.09 P= 0.003** | **F_1,74_= 21.34 P< 0.001** | **F_1,73_= 4.54 P= 0.036** |
| Decomposer species # | **F_3,74_= 4.14 P= 0.009** | **F_1,74_= 8.70 P= 0.004** | F_1,74_= 0.32 P= 0.571 |
| Decomposition | **F_3,74_= 4.86 P= 0.004** | **F_1,74_= 19.88 P< 0.001** | F_1,74_= 0.44 P= 0.507 |
| Herbivore abundance | **F_3,72_= 4.87 P= 0.004** | **F_1,72_= 33.26 P< 0.001** | F_1,72_= 0.98 P= 0.326 |
| Herbivore species # | **F_3,72_= 2.81 P = 0.045** | **F_1,72_= 23.38 P < 0.001** | F_1,72_= 0.22 P= 0.643 |
| Herbivory rate | **F_3,72_= 2.30 P= 0.084** | **F_1,72_= 8.40 P= 0.005** | F_1,72_= 2.08 P= 0.154 |
