# Supplementary material for: Plant Diversity Impacts Decomposition and Herbivory via Changes in Aboveground Arthropods
Source: PLoS One. 2014 Sep 16;9(9):e106529. doi: 10.1371/journal.pone.0106529 (PMC4165753; doi:10.1371/journal.pone.0106529)
Supplement: Table S2 — Results of the path analysis linking plants, decomposers and decomposition. (DOCX) [file pone.0106529.s004.docx]

**Table S2:** Results of the path analysis linking plants, decomposers and decomposition.

The table shows unstandardized path coefficients (estimate), standard error of regression weight (S.E.), the critical value for regression weight (C.R.) and level of significance for regression weight (*P*) for effects of plant diversity (log transformed), aboveground biomass (g m^-^², square root transformed), and C:N ratio of plant aboveground biomass on abundances (log transformed) and species richness of decomposers (log transformed) and decomposition (mg mg^-1^ d^-1^). Significant paths are given in bold.

| **Path** | | | **Estimate** | **S.E.** | **C.R.** | **P** |
| --- | --- | --- | --- | --- | --- | --- |
| **Decomposition (Fig. 2a)** | | | | | | |
| **Plant C:N ratio** | **←** | **Plant diversity** | **1.86** | **0.49** | **3.77** | **<0.001** |
| **Plant biomass** | **←** | **Plant diversity** | **3.12** | **0.40** | **7.74** | **<0.001** |
| Decomposer abundance | ← | Plant diversity | 0.13 | 0.11 | 1.13 | 0.261 |
| Decomposer abundance | ← | Plant C:N ratio | 0.02 | 0.02 | 0.94 | 0.349 |
| **Decomposer abundance** | **←** | **Plant biomass** | **0.06** | **0.02** | **2.75** | **0.006** |
| **Decomposer species #** | **←** | **Decomposer abundance** | **0.60** | **0.21** | **2.85** | **0.004** |
| Decomposer species # | ← | Plant diversity | >0.01 | 0.21 | 0.02 | 0.986 |
| Decomposer species # | ← | Plant biomass | 0.08 | 0.05 | 1.75 | 0.079 |
| Decomposition | ← | Decomposer abundance | >0.01 | >0.01 | 0.88 | 0.382 |
| **Decomposition** | **←** | **Decomposer species #** | **>0.01** | **>0.01** | **2.17** | **0.030** |
| **Decomposition** | **←** | **Plant diversity** | **>0.01** | **>0.01** | **3.01** | **0.003** |
