# Supplementary material for: Plant Diversity Impacts Decomposition and Herbivory via Changes in Aboveground Arthropods
Source: PLoS One. 2014 Sep 16;9(9):e106529. doi: 10.1371/journal.pone.0106529 (PMC4165753; doi:10.1371/journal.pone.0106529)
Supplement: Table S3 — Standardized total effects of the structural equation model analysing plant diversity effects on decomposition. (DOCX) [file pone.0106529.s005.docx]

**Table S3:** Standardized total effects (sum of direct and indirect effects) of the structural equation model analysing plant diversity effects on decomposition given as standardised path coefficients. Plant diversity and decomposer abundance were log transformed, and aboveground biomass (g m^-^²) square root transformed.

|  | ***Plant species***  ***richness*** | ***Plant***  ***biomass*** | ***Plant***  ***C:N ratio*** | ***Decomposer***  ***abundance*** | ***Decomposer***  ***species #*** |
| --- | --- | --- | --- | --- | --- |
| **Plant biomass** | 0.66 | - | - | - | - |
| **Plant C:N ratio** | 0.39 | 0 | - | - | - |
| **Decomposer abundance** | 0.43 | 0.36 | 0.10 | - | - |
| **Decomposer species #** | 0.30 | 0.36 | 0.03 | 0.33 | - |
| **Decomposition** | 0.44 | 0.12 | 0.02 | 0.18 | 0.24 |
