# Supplementary material for: Plant Diversity Impacts Decomposition and Herbivory via Changes in Aboveground Arthropods
Source: PLoS One. 2014 Sep 16;9(9):e106529. doi: 10.1371/journal.pone.0106529 (PMC4165753; doi:10.1371/journal.pone.0106529)
Supplement: Table S4 — Results of the path analysis linking plants, herbivores and herbivory rate. (DOCX) [file pone.0106529.s006.docx]

**Table S4:** Results of the path analysis linking plants, herbivores and herbivory rate.

The table shows unstandardized path coefficients (estimate), standard error of regression weight (S.E.), the critical value for regression weight (C.R.) and level of significance for regression weight (*P*) for effects of plant diversity (log transformed), summer aboveground biomass (g m^-^², square root transformed), and C:N ratio of summer plant aboveground biomass on abundances (log transformed) and species richness of herbivores (log transformed) and herbivory rate (logit transformed). Significant paths are given in bold.

| **Path** | | | **Estimate** | **S.E.** | **C.R.** | **P** |
| --- | --- | --- | --- | --- | --- | --- |
| **Herbivory (Fig. 2b)** | | | | | | |
| **Plant C:N ratio** | **←** | **Plant diversity** | **2.46** | **0.46** | **5.34** | **<0.001** |
| **Plant biomass** | **←** | **Plant diversity** | **1.65** | **0.34** | **4.86** | **<0.001** |
| **Herbivore abundance** | **←** | **Plant diversity** | **0.25** | **0.08** | **2.98** | **0.003** |
| **Herbivore abundance** | **←** | **Plant C:N ratio** | **0.04** | **0.02** | **2.37** | **0.018** |
| Herbivore abundance | ← | Plant biomass | 0.01 | 0.02 | 0.45 | 0.650 |
| **Herbivore species #** | **←** | **Herbivore abundance** | **2.53** | **0.72** | **3.51** | **<0.001** |
| **Herbivore species #** | **←** | **Plant diversity** | **1.62** | **0.55** | **2.97** | **0.003** |
| Herbivore species # | ← | Plant biomass | -0.22 | 0.14 | -1.52 | 0.127 |
| **Herbivory rate** | **←** | **Herbivore abundance** | **0.31** | **0.12** | **2.50** | **0.012** |
| Herbivory rate | ← | Plant diversity | 0.04 | 0.08 | 0.51 | 0.613 |
| Herbivory rate | ← | Herbivore species # | 0.03 | 0.02 | 1.36 | 0.175 |
