# Supplementary material for: Plant Diversity Impacts Decomposition and Herbivory via Changes in Aboveground Arthropods
Source: PLoS One. 2014 Sep 16;9(9):e106529. doi: 10.1371/journal.pone.0106529 (PMC4165753; doi:10.1371/journal.pone.0106529)
Supplement: Table S5 — Standardized total effects of the structural equation model analysing plant diversity effects on herbivory rate. (DOCX) [file pone.0106529.s007.docx]

**Table S5:** Standardized total effects (sum of direct and indirect effects) of the structural equation model analysing plant diversity effects on herbivory rate given as standardised path coefficients. Plant diversity and herbivore abundance were log transformed, summer plant aboveground biomass (g m^-^²) square root transformed, and herbivory rate logit transformed.

|  | ***Plant species***  ***richness*** | ***Plant***  ***C:N ratio*** | ***Plant***  ***biomass*** | ***Herbivore***  ***abundance*** | ***Herbivore***  ***species #*** |
| --- | --- | --- | --- | --- | --- |
| **Plant C:N ratio** | 0.52 | - | - | - | - |
| **Plant biomass** | 0.48 | 0 | - | - | - |
| **Herbivore abundance** | 0.52 | 0.26 | 0.05 | - | - |
| **Herbivore species #** | 0.47 | 0.10 | -0.14 | 0.38 | - |
| **Herbivory rate** | 0.31 | 0.10 | -0.01 | 0.38 | 0.17 |
